# Risk of Interprosthetic Femur Fracture Is Associated with Implant Spacing—A Biomechanical Study

**DOI:** 10.3390/jcm12093095

**Published:** 2023-04-24

**Authors:** Mischa Mühling, Sabrina Sandriesser, Claudio Glowalla, Sven Herrmann, Peter Augat, Sven Hungerer

**Affiliations:** 1 Institute for Biomechanics, BG Unfallklinik Murnau, Prof. Küntscher Str. 8, 82418 Murnau, Germany; mischa.muehling@bgu-murnau.de (M.M.);; 2 Institute for Biomechanics, Paracelsus Medical University, Strubergasse 21, 5020 Salzburg, Austria; 3 Department of Arthroplasty, BG Unfallklinik Murnau, Prof. Küntscher Str. 8, 82418 Murnau, Germany

**Keywords:** interprosthetic fracture, kissing implants, total hip arthroplasty, total knee arthroplasty

## Abstract

Background: Ipsilateral revision surgeries of total hip or knee arthroplasties due to periprosthetic fractures or implant loosening are becoming more frequent in aging populations. Implants in revision arthroplasty usually require long anchoring stems. Depending on the residual distance between two adjacent knee and hip implants, we assume that the risk of interprosthetic fractures increases with a reduction in the interprosthetic distance. The aim of the current study was to investigate the maximum strain within the femoral shaft between two ipsilateral implants tips. Methods: A simplified physical model consisting of synthetic bone tubes and metallic implant cylinders was constructed and the surface strains were measured using digital image correlation. The strain distribution on the femoral shaft was analyzed in 3-point- and 4-point-bending scenarios. The physical model was transferred to a finite element model to parametrically investigate the effects of the interprosthetic distance and the cortical thickness on maximum strain. Strain patterns for all parametric combinations were compared to the reference strain pattern of the bone without implants. Results: The presence of an implant reduced principal strain values but resulted in distinct strain peaks at the locations of the implant tips. A reduced interprosthetic distance and thinner cortices resulted in strain peaks of up to 180% compared to the reference. At low cortical thicknesses, the strain peaks increased exponentially with a decrease in the interprosthetic distance. An increasing cortical thickness reduced the peak strains at the implant tips. Conclusions: A minimum interprosthetic distance of 10 mm seems to be crucial to avoid the accumulation of strain peaks caused by ipsilateral implant tips. Interprosthetic fracture management is more important in patients with reduced bone quality.

## 1. Introduction

The number of total hip arthroplasties (THA) and total knee arthroplasties (TKA) is increasing worldwide in industrial countries with an ageing population as there is a positive correlation of osteoarthritis with age [1,2]. As a result, the frequency of revision surgeries after THA or TKA is also increasing. The most common reasons for revision surgery are infection, implant loosening or periprosthetic fractures [3,4,5]. Revision surgery of hip and knee arthroplasty is often associated with longer anchoring stems and requires significant surgical expertise [6,7]. Problems arise in patients with ipsilateral adjacent hip and knee implants with only a short residual length between implants. The risk of interprosthetic fractures is high because of the reduced bone quality in elderly patients and is further elevated by implant rigidity [8]. Megaprostheses, such as total femur replacement, as alternative treatment options are associated with high rates of intraoperative morbidity, postoperative infection and dislocation [9,10]. Therefore, the preferred surgical strategy is to preserve the native bone and minimize the risk of interprosthetic fractures.

At least four factors contribute to the strength of the femoral bone in between two ipsilateral adjacent femoral implants: the distance between the two implants, the cortical thickness, the bone quality and the anchoring technique [11,12,13]. Tight interprosthetic distances are thought to lead to excessive strain on the bone between the two implant tips, increasing the risk of an interprosthetic fracture. Soenen et al. showed in a finite element study that the risk of fracture increased for interprosthetic distances smaller than 110 mm, but did not investigate implant distances less than 50 mm [12]. It has also been observed that a decreased cortical thickness and an increased medullary diameter are associated with the occurrence of interprosthetic fractures [11]. However, despite these associations, the clinical evidence for the technical implementation of ipsilateral adjacent femoral implants is sparse [13]. In particular, there is a lack of clinical and biomechanical studies investigating the effect of different interprosthetic distances on the resulting risk of interprosthetic fracture. A better understanding of the effect of interprosthetic spacing on the risk of interprosthetic fracture would be a critical step in improving patient care. Reliable biomechanical evidence on the relationship between interprosthetic spacing and fracture risk may lead to clinical recommendations for the correct intraoperative placement of the endoprosthesis or, in cases of fracture, to the correct placement of the osteosynthesis.

The purpose of the present study was to determine the effect of the interprosthetic distance between implants on periprosthetic fracture risk. As a surrogate marker of fracture risk, strain on the bone surface was investigated. We hypothesized that reducing the interprosthetic distance or decreasing the cortical thickness of the femur would increase the maximum strain in the femoral shaft.

## 2. Materials and Methods

To investigate the strain in the femoral shaft between two ipsilateral implant tips, a simplified physical model consisting of synthetic bone tubes and metallic implant cylinders was built. Subsequently, the physical model was transferred into a finite element model (FE model) to perform a parametric analysis of the effects of interprosthetic distance and cortical thickness on the maximum strain in the femoral shaft.

### 2.1. Biomechanical Model

As a bone substitute, an epoxy glass laminate tube (Kruelit 750, Krueger & Sohn GmbH, Landshut, Germany) with an outer diameter of 24 mm and a wall thickness of 2 mm was cut to a length of 300 mm. The Young’s modulus of the material was investigated in an axial compression test and found to be 20.47 GPa, which matches human cortical bone [14,15,16]. Two aluminum cylinders were lathe faced to a diameter of 19.95 mm and a length of 170 mm and represented simplified stems of intramedullary implants. Biomechanical tests were carried out on a mechanical testing machine (Zwick Z010, Zwick Roell, Ulm, Germany) using a 10 kN load cell (Serie K, accuracy 0.5, GTM Testing and Metrology, Bickenbach, Germany).

**Figure 1:**
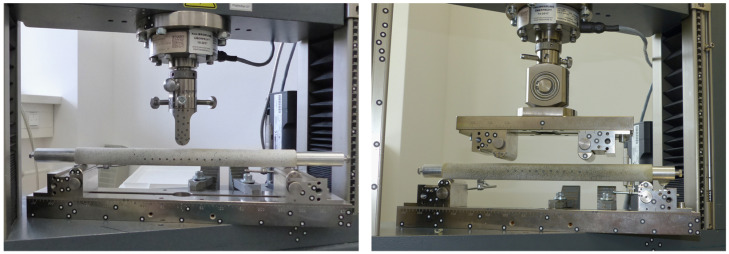
Experimental setup for quasi-static 3-point-bending (**left**) and 4-point-bending (**right**). The load cell is mounted on the machine actuator and a simple semicircular load applicator is used for 3-point-bending and two load applicators on a rocker were used for 4-point-bending.

To investigate realistic loading scenarios on the femoral shaft, such as a fall with lateral impact and load induced during activities of daily living, the constructs were tested under 3-point-bending (3PB) and 4-point-bending (4PB), respectively (Figure 1). The distance between the lower supports was 280 mm for both load cases. For 4PB, the upper supports were mounted on a rocker at a distance of 120 mm. The support pads were semicircular with a diameter of 10 mm. 

For each bending scenario, a separate bone tube was used and the aluminum cylinders were inserted symmetrically to the defined depth. In both setups the same interprosthetic distances were investigated: 0 mm, 5 mm, 10 mm, 20 mm, 30 mm, 40 mm, 50 mm, 60 mm, 80 mm, 100 mm and without any implants. The constructs were loaded in two consecutive ramps within its linear elastic region at a velocity of 50 N/s, up to a maximum load of 800 N for 3PB, resulting in a bending moment of 56 Nm, and a load of 1600 N for 4PB, resulting in a bending moment of 64 Nm. A preload of 2 N guaranteed reproducible reference conditions.

**Figure 2:**
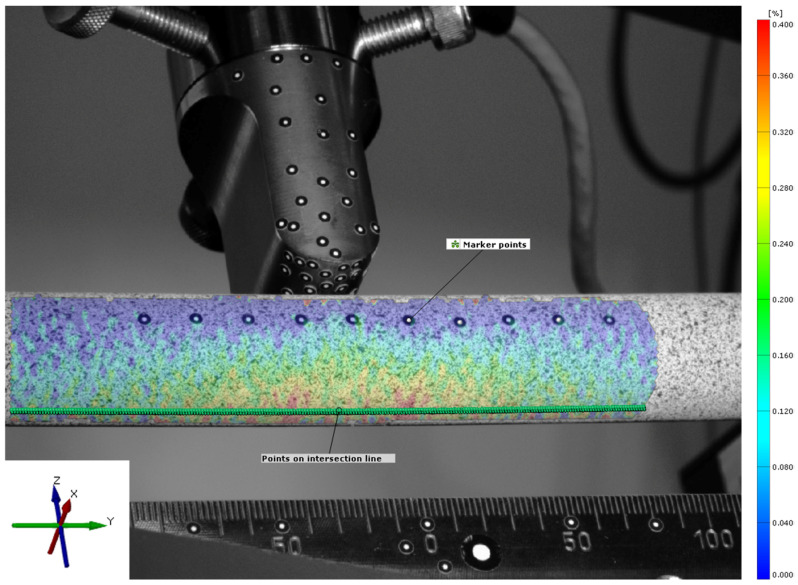
Strain measurement on the stochastic spray pattern based on points along the intersection line. The color coding provides information on the amount and distribution of the tensile strain from 0% strain (blue) to 0.4% strain (red). Attached marker points along the bone tube were used for analysis of the bending curve for FE model validation.

To investigate strains at the surface of the synthetic bone tubes, the tubes were sprayed with a stochastic pattern that was detected by an optical measurement system (ARAMIS 5M, GOM GmbH, Braunschweig, Germany). For validation of the FE model, ten additional marker points were attached along the tube to measure absolute deformation of the tube (Figure 2). Strain patterns were analyzed by digital image correlation at the maximum loading conditions (GOM Correlate Professional 2020, GOM GmbH, Braunschweig, Germany). The coordinate system was defined with the *y*-axis aligned along the axis of the bone tube and the *z*-axis aligned vertically in the direction of the machine actuator. The center of the coordinate system was placed in the center of the bone tube, directly below the load actuator. Only cortical strain on the tension surface of the tube, opposite from the load actuator, was analyzed. Therefore, virtual points were created along an intersection line on the surface. These points were placed at a distance of 0.5 mm and strains in the direction of the bone axis (*y*-axis) were evaluated over a total length of 110 mm.

### 2.2. Finite Element Model

The physical models of the tube and implants were replicated in ANSYS Design Modeler (ANSYS 2022 R1, Canonsburg, PA, USA). Static structural simulations were built with two variations: (a) 3- and 4-point-bending loading case and (b) with and without implants. The load applicator and bearings were modeled as rigid bodies at the same distances as in the experimental part. 

**Figure 3:**
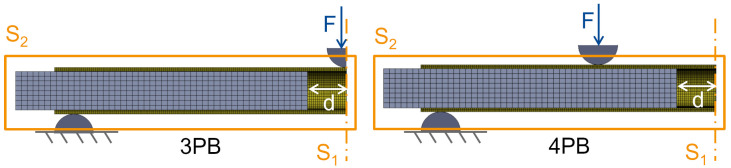
Mechanical specification of the FE model with additional information about the symmetry planes (S1 and S2), definition of the implant distance (d) and the applied force (F) in 3-point-bending (3PB) and 4-point-bending (4PB).

ANSYS Mechanical was used to build and calculate the non-linear FE simulations using an implicit solver. To reduce simulation time and resources, a quarter of the model was calculated using two symmetry planes to maintain the mechanical situation (Figure 3). A mesh convergency study resulted in 23,532 (with implant) and 20,882 (without implant) quadratic hexahedral elements. The material properties for simulation were applied according to the manufacturer’s information (Table 1). Contacts between the implant and the tube as well as the tube and bearings were modeled as frictional contacts with coefficients of 0.1 and 0.3, respectively. In the parametric analysis, the implant distance to the symmetry axis was varied between 0.5 mm and 60 mm to simulate interprosthetic distances between 1 mm and 120 mm. The thickness of the bone tube was varied between 2, 4 and 6 mm to analyze the effect of varying the cortical bone thickness.

**Figure 4:**
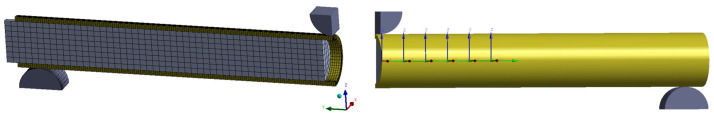
Isometric mesh model (**left**) and 6 points of interests used for comparison with the mechanical tests to validate the bending curve (**right**) of the FE model.

Validation was performed by comparing the experimental data with the simulation results. The deformation of attached marker points in the middle of the tube, the strain at the tensile site of the tube at the center, 40 mm off the center and at the implant location were analyzed using the relative deformation of the facets on the spray pattern (Figure 4). The experimental and simulation data were analyzed using a linear fit and Pearson’s correlation to judge the quality of prediction. Additionally, the principal strain pattern over the tensile area of the tube was compared at a 20 mm implant distance between experiment and simulation. Data were analyzed and visualized and the root mean square error (RMSE) was calculated in Matlab (R2022b, The MathWorks, Portola Valley, CA, USA).

## 3. Results

**Figure 5:**
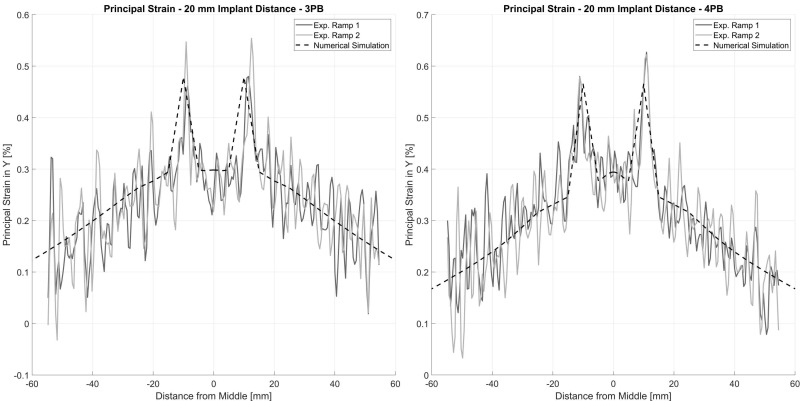
Principal strain for 20 mm interprosthetic distance for 3-point-bending (**left**) and 4-point-bending (**right**). The solid lines show the experimental data for two consecutive loading ramps and the dashed line represents the calculation from the FE model.

**Figure 6:**
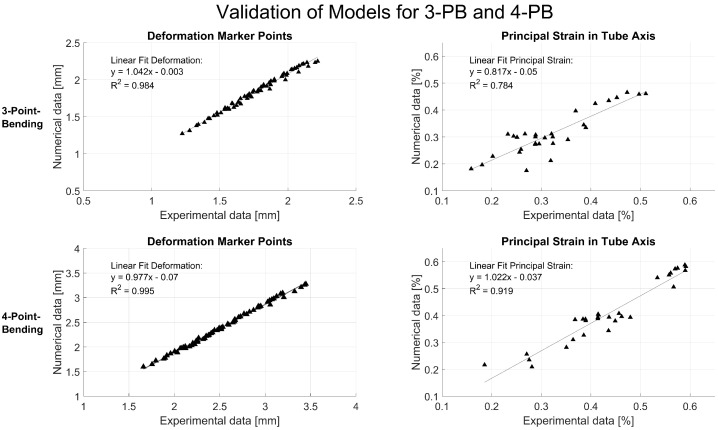
FE model validation over six marker points and principal strain along the tube axis. The model deformations were compared to the experimental data and analyzed using Pearson’s correlations.

Without an implant, the surface strains at the tensile site were 0.32% and 0.41% of the absolute principal strain for 3PB and 4PB, respectively. The presence of the intramedullary implants reduced the overall strain on the surface of the bone tube by about 0.05% of the absolute strain but generated distinct strain peaks at the respective positions of the implant tips. At the implant tips, the strain values were amplified by about 0.2% of the absolute strain. For an interprosthetic distance of 20 mm, peak strains of approximately 0.5% for 3PB and 0.6% for 4PB were identified (Figure 5). The strain patterns and the strain values measured in the experiment were well represented by the numerical calculation with the FE model. The validation showed very good correlations of the measured tube deformation (RMSE: 0.08 mm for 3PB and 0.13 mm for 4PB) and the local strain values (RMSE: 0.04% for 3PB and 4PB) with the respective simulated values (Figure 6).

**Figure 7:**
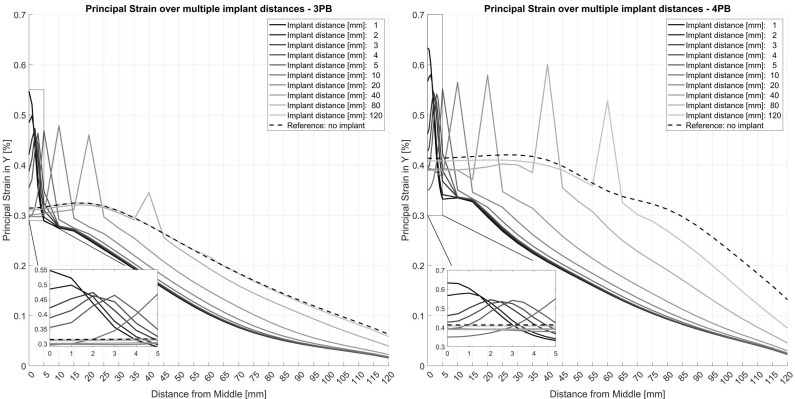
Principal strain analysis of different interprosthetic distances for a 2 mm cortical thickness in 3- and 4-point-bending. Due to the symmetry of the model, a distance of 20 mm represents an interprosthetic distance of 40 mm.

The FE model was first employed to assess the effect of the distance between the implant tips on the strain values of the tube. Overall, the presence of an implant reduced the principal strain values in the tube over its entire length. However, the change in material properties at the tip of the implant resulted in distinct peaks of the principal strain values of up to 0.55% for 3-point-bending and up to 0.63% for 4-point-bending (Figure 7). These peaks were consistently located at the respective positions of the implant tips. For 4-point-bending, these distinct peaks were found at all investigated implant distances. For 3-point-bending, the strain peak for 80 mm implant distance was reduced to 0.35% and no peak was detected at an implant distance of 120 mm. 

**Figure 8:**
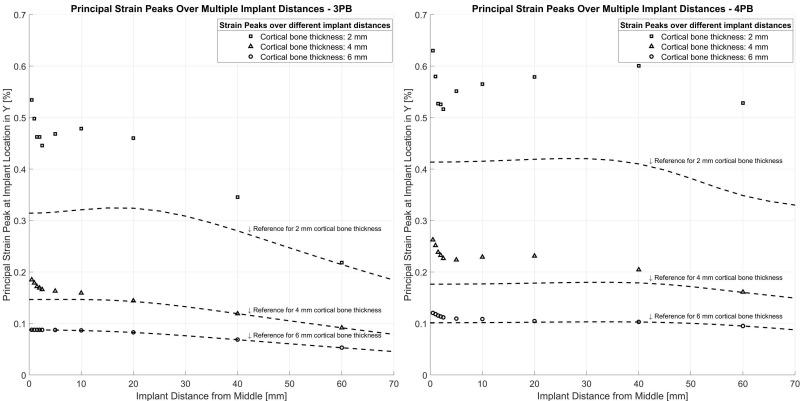
Principal strain peaks at implant position for different cortical bone thicknesses in 3- and 4-point-bending. Due to the symmetry of the model, a distance of 20 mm represents an interprosthetic distance of 40 mm.

The FE model was further employed to assess the effect of cortical thickness on the surface strain in the presence of an intramedullary implant (Figure 8). The peak strains generally decreased with increasing cortical thickness. The amplification of the strain at the locations of the implant tips was more pronounced with thinner cortices. For a cortical thickness of 6 mm, the strain amplification was less than 0.02% strain for 4-point-bending and almost indiscernible for 3-point-bending. For 3-point-bending, the peak strains remained at a low level of 0.1 to 0.2% for a 4 mm cortical thickness and increased up to 0.5% for a 2 mm cortical thickness. For 4-point-bending, the peak strains were between 0.2% and 0.3% for a 4 mm cortical thickness and up to 0.65% for a 2 mm cortical thickness.

## 4. Discussion

This study provides a valid finite element model to investigate the effect of interprosthetic distances between ipsilateral implants on the strain pattern in femoral shafts. Overall, the presence of an implant reduced the principal strain values in the femoral shaft over its entire length. However, distinct strain peaks were identified at the locations of the implant tips. Depending on the interprosthetic distance and cortical thickness, the strain was magnified by up to 80%. An increased cortical thickness reduced the peak strains at the implant tip position and had a greater effect on overall stiffness than the interprosthetic distance did.

For interprosthetic distances of less than 10 mm, the strain values increased exponentially due to accumulation of the strain peaks of the individual implant tips. This may be one of the reasons for bone failure around implant tips observed in clinical practice. This result is consistent with Sun et al., who described a shift in strain concentration in ipsilateral implants to the area between the two implants [17]. The strain peak pattern across the tube at a 10 mm interprosthetic distance and beyond was different for the two different loading cases. In the case of 3-point-bending, the overall strain values decreased, while in the case of 4-point-bending, the strain values remained relatively constant over the course of the tube. This phenomenon can be explained by the nature of the loading cases, where in 4-point-bending, the applied moment was constant between the load applicators. In contrast, in 3-point-bending, the applied moment decreases along the tube.

Soenen et al. found an increased fracture risk in 4-point-bending scenarios and suggested a minimal interprosthetic distance threshold of 110 mm. However, they did not test interprosthetic distances of less than 50 mm. Thus, the exponential strain peak effect that occurred in the present study at 10 mm or less was not found [12].

Another study by Walcher et al. investigated the effect of plate positioning in periprosthetic or interprosthetic femur fractures and found a strain increase on the bone with a decreasing overlap or gap in the implants. According to the authors, this might not be a similar biomechanical effect as the strain peak effect in the present study analyzing intramedullary implants [18]. Further investigations are necessary to substantiate or contradict this statement.

Clinically, an increase in fracture severity was found by Townsend et al. when a total hip prosthesis and a total knee prosthesis are present in one bone. In one third of the cases, the fracture occurred distal to the hip implant, resulting in unstable bending-type fractures more often than in a group that only had hip implants inserted [19]. These findings suggest a stress increasing effect of adjacent implants and their distances.

In a finite elements analysis study by Plausinis et al., the effect of interprosthetic spacing in the humerus was examined. They claimed that the stresses near the stem tips of the ipsilateral prostheses did not increase above the level seen in single implant cases. In their study, they used pure bending and torsional moments of 10 Nm with tubes of 1.5 mm and 3 mm cortical thicknesses [20]. In the present study, similar results were obtained with the 6 mm thick tube samples. This indicates that the occurrence of strain peaks in ipsilateral settings is strongly dependent on cortical thickness in relation to the amount and type of loading. Patients are postoperatively advised to bear their full weight if tolerated after 2 to 4 months, which increases the bending load on the femur. Thus, a higher load for the test setup seems more suitable [6,21]. Weiser et al. also demonstrated the importance of cortical thickness but rejected a critical effect of interprosthetic distance on strain amplification between implants [22]. In the present study, a constant increase in strain peaks was observed with decreasing cortical thickness. Bone quality is reported as one major factor for interprosthetic fracture risk in the literature, confirming the findings of this study [8,11,23]. The present results suggest that interprosthetic distance has an important effect on the strain pattern when the cortical thickness is 4 mm or less.

The constructs were mechanically loaded in 3-point- and 4-point-bending to cover clinically relevant loading scenarios. Three-point-bending was thought to mimic loading during an unintentional loading event, such as a fall onto the side or onto an obstacle. Four-point-bending, which produces a more homogeneous bending moment along the femoral shaft, was thought to mimic the loading that occurs during walking due to ground reaction and muscle forces. Strain magnification at the implant tips was similar for both loading scenarios when the interprosthetic distance was 40 mm or less. For larger interprosthetic distances, the strain magnification was less pronounced for 3-point-bending compared to 4-point-bending.

The limitations of the study include the simplified representation of a femoral shaft by a cylindrical bone substitute. Human cadaveric specimens demonstrate inter-specimen variability, such as cortical thickness, geometry and mechanical properties. On the other hand, bone surrogates with human anatomy eliminate this problem, but by anatomical nature, the cortical thickness changes over the axial length of the bone [24]. Therefore, a parametrical analysis of the interprosthetic distance in these specimens is not possible to be isolated but is combined with the parameter of cortical thickness. Since the material properties were comparable to those of human bone, the synthetic tube provides a good alternative and allowed to investigate the effect of strain caused by ipsilateral implants. The material was chosen because it represents a homogenous thickness of cortical bone over the tube length; therefore, it is well suited for a parametrical analysis of interprosthetic distance with constant geometric conditions. Furthermore, implant stems for hip and knee arthroplasties typically have conical tips to facilitate easier implant insertion and to reduce strain peaks. The strain values were not evaluated continuously, but at intervals of 0.5 mm for distances below 5 mm distance and in 5 mm intervals for distances larger than 5 mm. Therefore, the spatial resolution could have affected the strain data, but the differences are expected to be minor. In a clinical setting, the implant stems derive their stability through press-fit anchorage or embedding in bone cement. Although the outer diameter of the aluminum cylinders was close to the inner diameter of the tubes, the fit was not perfectly tight to allow for a reproducible and precise manipulation of the implant position. Loose stems have previously been shown to produce larger strain peaks than fixed or embedded stems [25]. Therefore, our boundary conditions without bonding between the implant stem and the outer bone tube may have overestimated the absolute amount of strain magnification. 

## 5. Conclusions

The findings from this study suggest a minimum interprosthetic distance of 10 mm to avoid the accumulation of strain peaks caused by adjacent implant tips. Strain amplification occurred at reduced cortical thicknesses of 4 mm and 2 mm but was not detectable at 6 mm. Therefore, careful interprosthetic management becomes more important in patients with reduced bone quality. Additional clinical and biomechanical studies are needed to further analyze the relationship between interprosthetic distance and strain amplification in the femoral shaft for different implant fixations and to develop an index for interprosthetic fracture risk assessments.

## Figures and Tables

**Table 1:** Material properties of the bone tube and the aluminum cylinder.

Part	Material	Young’s Modulus (MPa)	Poisson’s Ratio
Bone tube	HGW 2735.4 (DIN 7735) EP GC 22 (EN 61212)	20,470	0.18
Implant cylinder	Aluminum Alloy	66,530	0.33

## Data Availability

Data relating to this study are available upon reasonable request.
